# A Journey in Standard Development: The Core Manufacturing Simulation Data (CMSD) Information Model

**DOI:** 10.6028/jres.120.016

**Published:** 2015-11-17

**Authors:** Yung-Tsun Tina Lee

**Affiliations:** National Institute of Standards and Technology, Gaithersburg, MD 20899

**Keywords:** CMSD, information model, interoperability, machine shop, manufacturing, simulation, standard

## Abstract

This report documents a journey “from research to an approved standard” of a NIST-led standard development activity. That standard, Core Manufacturing Simulation Data (CMSD) information model, provides neutral structures for the efficient exchange of manufacturing data in a simulation environment. The model was standardized under the auspices of the international Simulation Interoperability Standards Organization (SISO). NIST started the research in 2001 and initiated the standardization effort in 2004. The CMSD standard was published in two SISO Products. In the first Product, the information model was defined in the Unified Modeling Language (UML) and published in 2010 as SISO-STD-008-2010. In the second Product, the information model was defined in Extensible Markup Language (XML) and published in 2013 as SISO-STD-008-01-2012. Both SISO-STD-008-2010 and SISO-STD-008-01-2012 are intended to be used together.

## 1. Background

In 2001, the Manufacturing Systems Integration Division (MSID, now SID – Systems Integration Division) of the National Institute Standards and Technology (NIST) Manufacturing Engineering Laboratory (MEL, now EL – Engineering Laboratory) began a project with a number of industrial partners and researchers. That project was designed to develop neutral formats for machine-shop data to facilitate simulation and modeling activities. A machine-shop information model, as a neutral interface format, was developed with support from both NIST’s Systems Integration for Manufacturing Applications (SIMA) Program [1] and the Software Engineering Institute’s (SEI) Technology Insertion Demonstration and Evaluation (TIDE) Program [2]. The SIMA Program supported NIST projects in applying information technologies and standards-based approaches to manufacturing software integration problems. The TIDE Program was sponsored by the Department of Defense (DoD) and SEI. TIDE was engaged in a number of projects with various small manufacturers in the Pittsburgh, Pennsylvania area. NIST carried out the technical work in collaboration with SEI, Carnegie Mellon University, Duquesne University, iTAC Software AG, and the Kurt J. Lesker Company (KJLC) [3].

KJLC is an international manufacturer and distributor of vacuum products and systems that target the research and industrial vacuum markets. KJLC manufactures complete, automatically-controlled vacuum systems; their special emphasis is on custom-designed, thin-film-deposition systems for research in alloys, semiconductors, superconductors, and optical and optoelectronics. At the time of the research, these systems were manufactured in a machine shop contained within the KJLC facility. Information from KJLC’s machine-shop operation was used to help define requirements for the simulation models and the data-interface specifications described in this report. Their facility was also used as a pilot site for testing and evaluating the simulation models, neutral data interfaces, and other software developed under the TIDE project.

The machine-shop information model was developed with two goals in mind. The first was to provide support for the integration of software applications at a pilot facility – KJLC’s machine shop. The second was to provide a more broad foundation for a new standard data interface for manufacturing simulators and possibly for other manufacturing software applications such as manufacturing execution systems and production scheduling systems. The modeling work started by gathering information requirements, accomplished primarily through visits to the KJLC manufacturing plant. Most of their shop-operation information was in paper format or recorded on whiteboards. KJLC was hopeful that the machine-shop information model would provide a sharable, stable, and organized structure of information requirements or knowledge, and offer great benefits to the company.

In 2003, Charles Mclean and Tina Lee completed an initial version of the machine-shop information model in two languages: Unified Modeling Language (UML) and Extensible Markup Language (XML). The information model continued to evolve based on the experience and feedback from KJLC’s implementations and others researchers involved in the effort. After two years of evolution, the document, which was called Shop Data Model and Interface Specification, was published by Charles McLean, Tina Lee, Guodong Shao, and Frank Riddick [4].

The Specification was tested in two case studies: KJLC’s prototype scheduling system and Boeing’s aircraft-wing-assembly simulation [5]. The Specification was used successfully to generate the correct interface data. These tests identified the need to facilitate implementations of, and to demonstrate the feasibility and capability of, the Specification. To address these needs, MSID researchers and industrial partners also helped to develop a machine-shop database model, a machine-shop data editor, and translators [6].

## 2. Technology Transfer – Applications Using the Machine-shop Information Model

More than a dozen technical papers related to the machine-shop information model have been published by MSID [5–21]. Topics of these papers included prototype implementation, reference architecture, data transfer strategy, and XML document validation.

During the time that the machine-shop information model was being developed and used in manufacturing simulation and integration research projects, it came to the attention of Philomena Zimmerman. She was an Associate Director at the Defense Modeling and Simulation Office (DMSO, now the Defense Modeling and Simulation Coordination Office) and DMSO liaison to the Simulation Interoperability Standards Organization (SISO) Executive Committee. In May 2003, Zimmerman contacted us about a potential joint effort between DMSO and NIST; she invited Charles McLean and Tina Lee to give a presentation at DMSO. As a result, Zimmerman recommended that NIST promote our machine-shop information model to SISO for standardization. The major reasons were 1) SISO is a recognized Sponsor Committee of the Institute of Electrical and Electronics Engineers (IEEE) Standards Association (SA); 2) SISO is a home for diverse modeling and simulation (M&S) communities; and 3) SISO is promoting the participation of and working to draw in manufacturing industry participants.

In April 2004, three NIST representatives met with the chairperson of the SISO Standards Activity Committee (SAC) to discuss the possibility of standardizing a specification based on the machine-shop information model. The Committee gave very positive responses and provided information necessary to create a SISO Product Development Group (PDG), the SISO mechanism through which standards are developed, balloted, and approved. With support from SISO management, NIST set its destination for international standardization.

## 3. Simulation Interoperability Standards Organization and SISO Products

SISO is an international organization dedicated to the promotion of M&S interoperability and reuse for the benefit of a broad range of M&S communities that include developers, procurers, and users worldwide [22]. The SISO SAC develops and supports M&S standards – both independently and in conjunction with other organizations. SISO is recognized as a Standards Development Organization (SDO) by the North Atlantic Treaty Organization (NATO) and as a Standards Sponsor by IEEE SA. SISO is a Category C Liaison Organization with the Joint Technical Committee 1 (JTC 1) of the International Organization for Standardization (ISO) and the International Electrotechnical Commission (IEC), i.e., ISO/IEC JTC 1. ISO/IEC JTC 1 was created in 1987 to ensure interoperability of standards related to information technology created by technical committees of ISO or IEC.

SISO Products are categorized into two tracks: balloted and un-balloted [23]. Un-balloted Products are reference and administrative products. For simplification, the term “SISO Products” is used to mean balloted SISO Standards Products for the rest of this report. The balloting of a SISO Product is required for final approval by the SISO SAC and SISO Executive Committee (EXCOM). SISO PDGs are formed to develop SISO Products and SISO Product Support Groups (PSGs) are formed to support approved SISO Products. There are four steps in developing a SISO Balloted Product: *Activity approval*, *Product development*, *Product balloting*, and *Product approval* [23].

*Activity approval* includes submitting and approving the product nomination (PN), i.e., a proposal. A PN describes needs, maturity, planned testing, proposed schedule, and candidate developer volunteers. The PN approval process includes a thirty-day review by the SISO community and approvals from both the SAC and EXCOM. Once the PN has been approved, a new PDG is chartered and product development begins.
*Product development* includes forming the PDG, conducting PDG meetings, developing the product, commenting on the draft product, and resolving the comments. A PDG is composed of SISO members interested in the product area and at least one Drafting Group, which produces the product or product components. The draft product, prepared by the Drafting Group(s), is based on either new original work or pre-existing work begun by other individuals or organizations. At various times during development, draft products are provided to the PDG membership for review and comment. Once the PDG has completed the product development step, the PDG presents the product to the SAC for approval to enter the ballot product phase, i.e., to begin balloting.
*Product balloting* includes establishing a circulation package, forming a ballot group, conducting the ballot, resolving the comments, and conducting re-circulation ballots as required. A circulation package consists of the product, meeting minutes, comment resolution artifacts, and relevant supporting material for major technical revisions. An invitation to join the ballot group is open to all interested individuals including non-SISO members; however, non-SISO members must join SISO to be included in the ballot group. A balanced ballot group that includes members from different organizations and technical areas is important to ensure community consensus in a SISO Product. The ballot group shall be balanced using three different criteria — *Representation*, *Organization*, and *Interest*.
  - *Representation*: Three categories exist for *Representation*: commercial, government, and academic. No *Representation* category shall exceed 75 % of the ballot group and each category shall be at least 10 % of the ballot group.
  - *Organization*: No single organization shall exceed 25 % of a *Representation* category.
  - *Interest*: Three categories exist for *Interest*: user, developer, and general interest. No *Interest* category shall exceed 50 % of the ballot group.
The ballot period for an initial ballot is between 30 and 60 days. During the ballot period, each member of the ballot group has the opportunity to accept or reject the product and to submit comments. For a product ballot to be valid, at least 75 % of the ballots must be returned. Also, for a product ballot to be successful, at least 75 % of all the ballots returned must accept the standard product. The PDG shall resolve all of the comments received. A re-circulation ballot is conducted if 1) the previous product ballot is unsuccessful and 2) the PDG and SAC agree that the balloting process should continue. The ballot period for a re-circulation product ballot is 30 days. When the balloting is complete, the product is ready for final approval by the SAC and EXCOM.
*Product approval* includes submitting the product-for-approval package and approving the product. A product approval package consists of the product nomination, final product draft, ballot results, and PDG activity documentation (e.g., PDG meeting minutes and pilot implementations.) The SAC is responsible for approving the product developed by the PDG and recommending a final disposition of the product to the EXCOM. This review may take 4 to 6 weeks. The EXCOM is responsible for the final approval of all products that will carry the SISO label.

Once the SISO Product is approved, it is ready for publication to the SISO community. In addition, all SISO-sponsored balloted products shall have a PSG established to provide continuity for the product. For ensuring credibility and integrity, SISO offers a mechanism for the periodic review of approved products with a focus on the maintenance of these products.

## 4. CMSD Standardization

This section presents an overview of CMSD and its associated standardization activities.

### 4.1 An Overview of CMSD

Currently, there are standards that address manufacturing data related to production operations, such as ISA-95 [24] and Open Applications Group Integration Specification (OAGi) [25]. CMSD and these standards all offer some interoperability solutions for data exchange between production-related software tools. However, only CMSD provides a means to specify information about the stochastic characteristics of production processes using probability distributions. This unique feature enables CMSD be used in, and exchanged among, discrete event and other types of simulation models of manufacturing operations. This feature has been critical for integrating different discrete event simulation technologies.

Before CMSD, there were no neutral data formats for storing the manufacturing data needed to develop and run simulation models. CMSD’s neutral structure represents the concepts, relationships, constraints, and rules that define the semantics of the “core” elements for that data. The advantage of using a neutral structure is that it can enable the creation of organized, self-consistent collections of information that can be reused by different applications without regard to proprietary license or intellectual property issues. In addition, CMSD facilitates the integration of simulation software with other manufacturing applications.

CMSD defines an information model in two commonly used modeling methods: UML and XML. UML is a graphical model representation and XML is a tag-based format for machine interpretable structured documents. Both languages can provide representations of the same information; in fact, the CMSD-XML [26] is mapped from the CMSD-UML [27]. CMSD-XML is not only useful for documentation but also useful for validation and process automation. The major CMSD entities and their definitions include bill of materials, calendar, distribution, inventory, job, layout, order, part, process plan, resource, and schedule. NIST made initial contact with SISO SAC in April 2004. Subsequently, they submitted a PN or proposal to SISO for establishing a PDG to develop a CMSD-type standard based on the NIST machine-shop information model. The proposal explained how CMSD 1) captured and described the characteristics of, and relationships among, the core manufacturing entities that define shop-floor operations and 2) would enable greater integration and simpler data exchange for manufacturing simulations and other manufacturing applications. The CMSD PDG was officially established in September 2004. Three NIST employees were on its leadership team and Peggy Gravitz (formerly) of AEgis Technologies and Mike Burnette of Joint Forces Command were appointed the Technical Area Directors of CMSD-UML and CMSD-XML, respectively.

Mapping an information model built in UML to its “mirror image” in XML can normally be done in a straightforward manner, with the help of a schema language. However, no existing schema language for XML could support all of the complex interrelationships between the entities defined by the CMSD-UML model. The PDG eventually followed a two-language approach: using REgular LAnguage for XML Next Generation (RELAX NG), which is a grammar-based language, and Schematron, which is a rule-based language, to accomplish the mapping task [28].

The CMSD specification was published in two SISO Products: CMSD-UML was officially published on September 20, 2010 as SISO-STD-008-2010 [27] and CMSD-XML was officially published on January 22, 2013 as SISO-STD-008-01-2012 [26]. Both SISO-STD-008-2010 and SISO-STD-008-01-2012 are intended to be used together.

For brevity, the rest of this report focuses on the standardization of CMSD-UML and leaves out CMSD-XML. This is because the major effort of developing the CMSD family of products was on CMSD-UML. Modeling CMSD-UML involved requirements analysis, modeling design, and case studies while CMSD-XML involved no additional requirements analysis and much less modeling design. Additionally, the case studies involving CMSD-XML were based on the existing case studies of CMSD-UML.

### 4.2 Technical Work

This section describes the development of the CMSD-UML model. The prototype development for CMSD testing is also discussed.

#### 4.2.1 Specifications Development

Extending the original machine-shop information model to the CMSD information model was not a simple process. This is because the original model was focused on one manufacturer and CMSD needed a much broader scope. That scope was to support the exchange of information between manufacturing-oriented simulations and other applications in manufacturing domains such as process planning, scheduling, inventory management, production management, and plant layout. While the manufacturing sector was the primary target, DoD’s interests and needs were taken into account from the beginning. Consequently, the participants of CMSD development included industrial users, military users, academic researchers, and software vendors. This meant that extending the original model to a UML model still required a major technical revision.

That extension was performed by the CMSD Drafting Group, led by NIST’s co-editors Frank Riddick and Tina Lee. The group started by gathering detailed requirements from literature reviews, site visits, domain-expert interviews, needs analysis, operational mission-requirements analysis, and trade-off analyses. The CMSD-UML document went through three major revisions before it was reviewed by the PDG membership in February 2009. Two more revisions were made based on the comments received from the ballot group and final reviews from the SAC and EXCOM. The final version, SISO-STD-008-2010, was published in September 2010.

#### 4.2.2 CMSD Validation

The content of CMSD and its feasibility for use as a data-exchange and application-integration mechanism called for pilot implementation tests. Industry use cases were often hard to acquire. In collaboration with industrial and academic partners, several prototype applications using initial or draft CMSD standards were developed for validation tests. Prototype development based on CMSD was undertaken to ensure that the information model was sufficiently detailed to describe fully the data needs of simulation applications. The prototypes were also used to provide evidence that CMSD was mature enough to warrant standardization.

One of the pilot implementations was performed under the Swedish research project, “Factory Analyses in Conceptual Phases Using Simulation” [28, 29]. That project’s purpose was to represent a detailed virtual model, including reusable objects and generic solutions, of a paint shop at a major automobile factory plant in Sweden. The model was created using a commercial simulation tool, Enterprise Dynamics^1^. In this study, CMSD was used to enable data collection of resources and work processes in the conceptual stages of production development programs. As a result, some discrepancies between CMSD data and Enterprise Dynamics input data were identified. One discrepancy was that the draft version of CMSD, upon which this prototype was based, did not directly support material handling equipment, such as conveyors and elevators. Also, that version of CMSD included mean-time-between-failure and mean-time-to-repair as attributes for resource definition but it did not include mean-time-to-failure and mean-down-time. The SISO CMSD PDG resolved the issue in the final version of CMSD by introducing the ability to represent user-defined attributes.

More case study results are documented in the technical paper, “Core Manufacturing Simulation Data – a Manufacturing Simulation integration Standard: Overview and Case Studies” [30].

### 4.3 Non-Technical Efforts

The biggest challenge during CMSD balloting was to establish the CMSD-UML ballot group. The difficulty was due to SISO’s ballot group balancing criteria — *Representation*, *Organization*, and *Interest* that is described in Sec. 3. The NIST leadership team successfully formed the balanced CMSD-UML ballot group of 42 members in early December 2009. With the ballot group formed, the PDG was able to start the balloting process; the ballot period was from December 16, 2009 to February 13, 2010.

The initial ballot was successful; no re-circulation ballot was needed. Of the 42 members of the ballot group, CMSD-UML had 34 “accept” votes and 8 “accept with comment” votes. No ballot group members voted to reject. Finally, CMSD-UML was officially approved by the EXCOM. The announcement of this approval was made at SISO’s Fall Simulation Integration Workshop’s Plenary Session held on September 21, 2010.

### 4.4 Transition from Development to Support

The CMSD PDG conducted its kick-off meeting at the 2004 Fall Simulation Interoperability Workshop. Work progressed through September 2010 when the PDG published its first SISO Product, SISO-STD-008-2010. The PDG’s second SISO Product, SISO-STD-008-01-2012, was finalized in 2012 and published in 2013. Since the CMSD PDG completed its work and there were no plans to develop additional SISO Products at that time, the PDG ceased operations and transitioned to the CMSD PSG in 2013. The PSG operates as a focused, task-organized group concentrating on the support of the SISO CMSD products. The group also serves as the central point for the interpretation of the product language. This means that it is responsible for accepting, developing, and maintaining problem/change reports to support future product revisions. In 2015, the PSG will conduct a required periodic review to make a recommendation whether to reaffirm, revise, or withdraw SISO-STD-008-2010.

## 5. Technology Transfer – Applications Using CMSD Standards

A literature review showed that in recent years many organizations including NIST have applied CMSD, both in draft and in final form, in various application domains where simulation has been involved [29–62]. The application domains where CMSD has been applied include:

- Supply Chain [32]
- Production engineering [40, 43, 45, 49]
- Design and planning [50, 57, 58]
- Sustainable Manufacturing [38, 41, 42, 44, 46, 47, 48, 51, 52, 55, 56, 59]
- Construction [60]

Many different commercial simulation systems were used in these efforts, including ExtendSim V8, 3DCreate, Plant Simulation, Enterprise Dynamics, Arena, and QUeuing Event Simulation Tool (QUEST); this demonstrates CMSD’s use as an interoperable representation for manufacturing simulation data.

While these efforts focused on developing solutions for manufacturing problems, international research efforts have also focused on the development of CMSD support methodologies or tools [35, 37, 49, 51, 53, 54, 61, 62].

## 6. Timeline

Table 1 below presents a timeline of the journey. It lists three major tasks, the major milestones of each task, and their corresponding time information.

## 7. Conclusion and Lessons Learned

This report described the process of developing the CMSD standards under the auspices of SISO, a Sponsor Committee of IEEE, and NATO-recognized SDO. CMSD grew out of a successful, long-term, NIST-led collaboration. The standards enable engineers to import and export many different types of factory data into computer simulations consistently and unambiguously. The CMSD standards can potentially make M&S capabilities more practical and cost-effective for a much broader spectrum of manufacturers. The standard has been applied both nationally and internationally to several application domains such as production engineering, construction, and sustainable manufacturing. Lessons learned from this experience include:

- Carefully select the SDO. Look into the following items:
  ○ Is the subject area of the proposed standard closely related to the scope of the SDO named?
  ○ Does the SDO have well-defined standard development guidelines?
  ○ Is the expected standard-development duration within a reasonable time frame to be of value to the stakeholder(s)?
  ○ Does the SDO membership have the technical expertise that the proposed standard needs?
- Engage candidate volunteers, including software vendors, researchers, and potential users, starting from the early stage.
- Engage the industry that the proposed standard is intended to support.
- Perform thorough needs and requirements analyses.
- Plan early for acquiring industrial use cases and datasets.
- Develop a number of test implementations before the final standard is released.
- Get commitment from upper management for time and labor.
- If filling a leadership role, be prepared for unexpected work load; volunteer committee members might come and go.
- Closely follow work progress for each process step required by the SDO.
- Promote the research and resulting specification early and prior to its adoption as a standard.

This journey of twelve years, from basic research to an official standard, was quite an experience.

## Figures and Tables

**Table 1:** The timeline of the CMSD standard development.

Task		Milestone		Reference
Title	Period	Subject	Time	
Research and Development	2001–2007	Machine-shop information model	6/2003	
		Machine-shop-information-model specification	1/2005	[4]
		MSID’s technical publications	2002–2009	[5–21]
Standardization	2004–2013	Proposal	5/2004	
		Product Development Group formed	9/2004	
		Initial CMSD-UML version	2/2009	
		CMSD-UML balloting	2/2010	
		CMSD-UML approved standard	9/2010	[27]
		CMSD-XML balloting	3/2012	
		CMSD-XML approved standard	1/2013	[26]
		Product Development Group dissolved	3/2013	
Use and Support	2007-present	Product Support Group formed	2/2014	
		Technical publications	2007–2013	[28–62]

## Footnotes

1 Commercial equipment, instruments, or materials are identified only in order to adequately specify certain procedures. In no case does such identification imply recommendation or endorsement by the National Institute of Standards and Technology, nor does it imply that the products identified are necessarily the best available for the purpose.
